# The public health benefit and burden of mass drug administration programs in Vietnamese schoolchildren: Impact of mebendazole

**DOI:** 10.1371/journal.pntd.0006954

**Published:** 2018-11-12

**Authors:** Sam Debaveye, Claudia Virginia Gonzalez Torres, Delphine De Smedt, Bert Heirman, Shane Kavanagh, Jo Dewulf

**Affiliations:** 1 Department of Green Chemistry and Technology, Ghent University, Campus Coupure, Ghent, Belgium; 2 Department of Public Health, Ghent University, Campus UZ, Ghent, Belgium; 3 Johnson & Johnson EHS&S, Janssen Pharmaceutica NV, Beerse, Belgium; 4 Health Economics, Janssen Pharmaceutica NV, Beerse, Belgium; University of Cambridge, UNITED KINGDOM

## Abstract

**Background:**

Mass anthelmintic drug administration is recommended in developing countries to address infection by soil-transmitted helminthiases (STH). We quantified the public health benefit of treatment with mebendazole in eight million Vietnamese children aged 5–14 years from 2006 to 2011. This was compared to the environmental impact of the pharmaceutical supply chain of mebendazole, as the resource use and emissions associated with pharmaceutical production can be associated with a public health burden, e.g. through emissions of fine particulate matter.

**Methodology:**

Through Markov modelling the disability due to STH was quantified for hookworm, *Ascaris lumbricoides* and *Trichuris trichiura*. For each worm type, four levels of intensity of infection were included: none, light, medium and heavy. The treatment effect on patients was quantified in Disability-Adjusted Life Years (DALYs). The public health burden induced by the pharmaceutical supply chain of mebendazole was quantified in DALYs through Life Cycle Assessment.

**Principal findings:**

Compared to ‘no treatment’, the modelled results of five-year treatment averted 116,587 DALYs (68% reduction) for the three worms combined and largely driven by *A*. *lumbricoides*. The main change in DALYs occurred in the first year of treatment, after which the results stabilized. The public health burden associated with the pharmaceutical supply chain was 6 DALYs.

**Conclusions:**

The public health benefit of the Mass Drug Administration (MDA) averted substantially more DALYs than those induced by the pharmaceutical supply chain. These results were verified in a sensitivity analysis. The starting prevalence for each worm was the most sensitive model parameter. This methodology is useful for policymakers interested in a holistic approach towards the public health performance of MDA programs, enveloping both the treatment benefit received by the patient and the public health burden associated with the resource consumption and environmental emissions of the pharmaceutical production and supply chain.

## Author summary

Millions of children from developing countries are infected by soil-transmitted helminthiases (STH), an infection of intestinal worms that cause abdominal pain, bad absorption of nutrients from food and a decrease in the amount of red blood cells. This disease can be treated with anthelmintic medication, such as mebendazole, that decreases the intensity of infection and leads to a public health benefit. Because reinfection often occurs within months, regular treatment (every six months) is advised. Given the number of people that are infected with this disease, numerous tablets are required each year to facilitate treatment. However, the industrial production of these tablets can have a negative effect on global human health, e.g. through emissions of fine particulate matter, which should be considered as a public health burden. Our findings suggest that the public health benefit of treating STH with anthelmintic medication is 18,035 times larger than the public health burden associated with pharmaceutical production. However, the conclusion that the health benefits for the patients outweigh the health damage due to resource use and emission from industry may not hold for every medical treatment, therefore we propose a more holistic evaluation of health care programmes, including a broader approach towards human health.

## Introduction

Every year, millions of children from developing countries receive medicines donated through the World Health Organization (WHO). In 2016, 1.3 billion tablets were shipped for the treatment of lymphatic filariasis, soil-transmitted helminthiases (STH) and schistosomiasis [1–3]. This study focuses on STH, which comprises four nematode infections: *Necator americanus*, *Ancylostoma duodenale*, *Ascaris lumbricoides* (roundworms) and *Trichuris trichiura* (whipworms). The first two are frequently combined and referred to as hookworms. In 2010, STH affected 1.45 billion people worldwide and is associated with high morbidity due to abdominal pain, anaemia and malabsorption of nutrients [4]. Children from the poorest developing countries are the most impacted by this disease, and 875 million children were reported to require annual treatment in 2012 [5].

In 2012, certain pharmaceutical companies, non-governmental organizations (NGOs), governments and banks signed the London Declaration on Neglected Tropical Diseases, committing to supply the necessary drugs to achieve control of STH by 2020 [6]. The global target is “*to eliminate morbidity due to soil-transmitted helminthiases in children by 2020*, *by regularly treating at least 75% of the children in endemic areas*” [7, 8]. In areas with high (>50%) prevalence of STH, the WHO recommends anthelmintic drug treatment every six months for school-aged children [7]. The effect of these treatments on the prevalence of STH is reported in multiple studies and the global public health impact of STH has been mapped in the Global Burden of Disease (GBD) [1, 9–12].

The pharmaceutical production, distribution and disposal of these anthelmintic drugs requires significant resources and causes emissions of hazardous compounds, which are associated with an effect on global human health, e.g. through emissions of fine particulate matter, which can be seen as a public health burden and quantified through Life Cycle Assessment (LCA) [13–15]. This environmental impact is currently not assessed along with the Mass Drug Administration (MDA) programs. However, the WHO recognizes that environmental factors (e.g. air pollution and Climate Change) are responsible for 22% of all global mortality and morbidity [16]. A holistic evaluation should compare both the public health benefit of MDA programs and the contribution to the public health burden attributable to the environment associated with the pharmaceutical production, distribution and disposal of the medicines [17].

The aim is to quantify and compare the public health benefit and burden for mebendazole MDA in Vietnam by using a common metric: the Disability-Adjusted Life Year (DALY) [18]. A DALY is equivalent to one healthy life year lost. This study focuses on Vietnam, building on a previously published model of STH prevalence progression after treatment with anthelmintic drugs, which are supplied to Vietnam through the MDA program [9, 19]. While multiple anthelmintic drugs are donated for the treatment of STH, data from the pharmaceutical supply chain was only collected for mebendazole. This demonstration study aims to provide a first insight on both the public health benefit for patients and public health burden attributable to the environment of MDA programs.

## Methods

The overall framework consists of two main parts. First, we discuss the simulation of the public health benefit of anthelmintic treatment with mebendazole every six months. The difference in public health morbidity associated with STH is quantified before and after the introduction of mebendazole MDA for eight million Vietnamese children aged 5–14 years for five years, reaching 80% coverage [20]. To do this we adopted a Markov model published by Montresor et al. [9, 19]. The model predicts the prevalence of hookworm, *A*. *lumbricoides* and *T*. *trichiura* on a yearly basis over a five year time horizon, which was combined with disability weights to calculate the population morbidity on a yearly basis [4].

Second, the public health burden of the pharmaceutical supply chain of mebendazole is quantified through an environmental LCA based on primary data. This cradle-to-grave assessment includes the resource use and emissions associated with the five supply chain stages: Active Pharmaceutical Ingredient (API) synthesis, tablet formulation, packaging, distribution and End-of-Life. The public health burden associated with the production of the 64 million tablets that are required for treatment in this study is a burden for the global population, as e.g. Climate Change is a global process. The functional unit, which is a measure of the function of the treatment to which the environmental impact can be related, is the same as in the first part [5].

Both the public health benefit and burden associated with mebendazole MDA are expressed in DALYs and compared to a theoretical counterfactual: the ‘no treatment’ group. This study focuses on calculating the disability attributable only to STH infection and treatment. Any disability from other causes was out of scope.

### Part I: Public health benefit of mebendazole MDA

The mebendazole MDA program, including treatment with mebendazole every six months for five years was compared with a ‘no treatment’ group where patients were not treated and worm prevalence and morbidity were assumed to stay constant over time on a population level [2].

The morbidity in the treated group was calculated on a yearly basis. The number of children infected by each separate worm type and intensity of infection at each year were multiplied with the disability associated with that specific infection. The prevalence and number of infected children were able to change over time, but the disability associated with each infection state is fixed.

#### STH prevalence in study population

The population under study was eight million Vietnamese children aged 5–14 that required anthelmintic treatment in 2006 [20]. This number is assumed to stay constant over 5 years on a population level. The STH prevalence before the introduction of MDA in 2006 (baseline prevalence) was obtained from van der Hoek et al. (2003) that determined the total STH prevalence for each worm type in 41,799 Vietnamese children aged 5–15 [21]. We then subdivided this into no infection, light, moderate and heavy infection intensity according to the WHO classification listed in Table 1 and produced estimated prevalence by STH type and severity shown in Table 2 [22, 23].

**Table 1:** Classes of soil-transmitted helminthiases (STH) infection based on the number of eggs per gram (epg) in a human stool sample [22].

	No infection (epg)	Light infection (epg)	Moderate infection (epg)	Heavy infection (epg)
Hookworm	0	1–1,999	2,000–3,999	≥4,000
*A*. *lumbricoides*	0	1–4,999	5,000–49,999	≥50,000
*T*. *trichiura*	0	1–999	1,000–9,999	≥10,000

**Table 2:** Soil-transmitted helminthiases (STH) prevalence for eight million Vietnamese children subdivided in light, moderate and heavy infection before mebendazole MDA [23, 24].

	Total prevalence		Light infection		Moderate infection		Heavy infection	
	%	Pop.	%	Pop.	%	Pop.	%	Pop.
Hookworm	24.60	1,968,000	23.81	1,904,512	0.67	53,648	0.12	9,840
*A*. *lumbricoides*	62.60	5,007,999	37.03	2,962,632	22.59	1,807,387	2.97	237,980
*T*. *trichiura*	26.00	2,080,000	22.70	1,816,048	3.23	258,336	0.07	5,616

#### Markov model

A published Markov model developed to monitor MDA programs for school-aged children was adopted to simulate the estimated reduction in STH prevalence due to mebendazole MDA [9, 19]. The model we adopted was Transition Probability Matrix Set (TPMS) 4 from the Supplementary Material of Montresor et al. (2016), representing the treatment of 1324 school-aged children with mebendazole every 12 months (Table A in S1 Text). The rationale for the model choice is outlined in S1 Text, page 2. As we aim to model the effect of treatment with mebendazole every six months, as recommended by the WHO when STH prevalence is higher than 50% and which was actually (partly) implemented in Vietnam, the results of TPMS 4 are considered conservative [7, 20].

The model timeframe for the simulation started from the situation in Vietnam in 2006 with a cycle length of one year and a five year time horizon, which was considered sufficiently long to capture all health effects, since the reduction in worm burden is already visible within 14 days and mortality was not considered [25]. Over five years, it is also possible to see the effects of reinfection, which often occurs within months [26].

**Fig 1:**
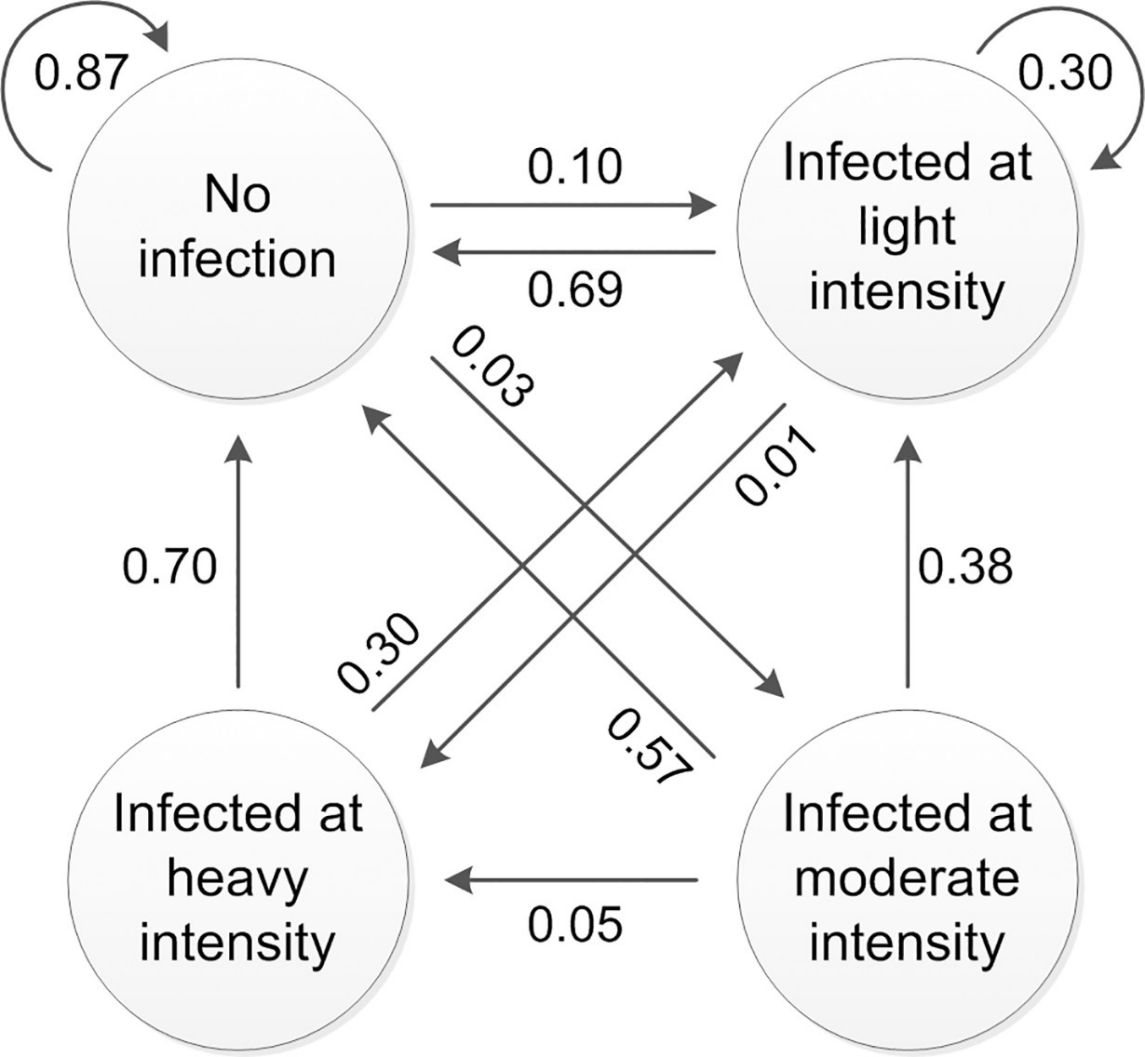
Markov model simulating the hookworm prevalence progression after each mebendazole treatment cycle of one year, adapted from Montresor et al. 2014 and Montresor et al. 2016 [19, 23].

The worm prevalence was calculated for each year and split up in no infection, light, moderate and heavy infection intensity. This subdivision enables a detailed disability quantification, as morbidity is highly dependent on worm burden, with heavy infection responsible for the largest share of disability. A fourth model state was defined as ‘no infection’. This structure, displayed in Fig 1 for the case of hookworm, allowed patients to move throughout the four model states each year as a result of pre-defined transition probabilities, representing treatment. A set of transition probabilities to go from one state to another exists for each worm type.

#### Model disability input

The disability associated with hookworm, *A*. *lumbricoides* and *T*. *trichiura* was estimated according to both the causes and weights of disability defined by the Institute for Health Metrics and Evaluation (IHME) GBD reports [4, 27].

For the purposes of the model we considered the following disability causes:

- Mild abdominopelvic problems, which occur in all cases of moderate infection.
- Symptomatic infection, which occurs in all cases of heavy infection for *A*. *lumbricoides* and *T*. *trichiura* but not for hookworm.
- Severe wasting, with country-specific prevalence, which occurs in all cases of heavy infection for hookworm, *A*. *lumbricoides* and *T*. *trichiura*.
- Anaemia, which is further split up into mild, moderate and severe anaemia. All intensities of anaemia are present for all intensities of hookworm infection. Anaemia is defined as a condition in which haemoglobin levels in the blood are below a certain age-dependent threshold, listed in Table 3 [28].

We carried out two literature reviews, subdivided into four sub-analyses, with the aim to support the assumptions regarding the disability inputs of anaemia and wasting in the model. The search terms and results can be found in Figure A, B, C, D and E in S1 Text.

**Table 3:** Anaemia thresholds based on haemoglobin level (g/l) [28].

	No anaemia	Mild anaemia	Moderate anaemia	Severe anaemia
Children 5–11 years of age	115 or higher	110–114	80–109	Lower than 80
Children 12–14 years of age	120 or higher	110–119	80–109	Lower than 80

Disability due to anaemia was only considered for hookworm, because intestinal blood loss due to STH infection is associated with this worm species [4]. Anaemia prevalence is influenced by the intensity of infection [29, 30]. The total prevalence of anaemia was subdivided in mild, moderate and severe anaemia. We carried out a first literature review with the aim to support the assumptions on both the total prevalence of anaemia associated with hookworm and the relative fractions of the subdivision into mild, moderate and severe anaemia (see Figure A, B, C and E in S1 Text).

Anaemia is caused by multiple factors, and only a fraction of the disability by anaemia can be allocated to hookworm and hence treated by deworming [31–33]. The base case percentage of anaemia that is attributable to hookworm was estimated to be 22% [34]. Although the assumption that deworming actually reduces the fraction of anaemia associated with hookworm was supported by the literature, a scenario analysis with data from multiple sources was performed due to the lack of agreement regarding this attributable fraction (see Table 4) [30, 35, 36].

**Table 4:** Scenarios on the percentage of anaemia attributable to hookworm.

	Anaemia percentage attributable to hookworm	Source
Scenario 1: most conservative	0.00%	
Scenario 2: after introduction of MDA in Vietnam	4.12%	[35]
Scenario 3: base case	22.00%	[34]

**Fig 2:**
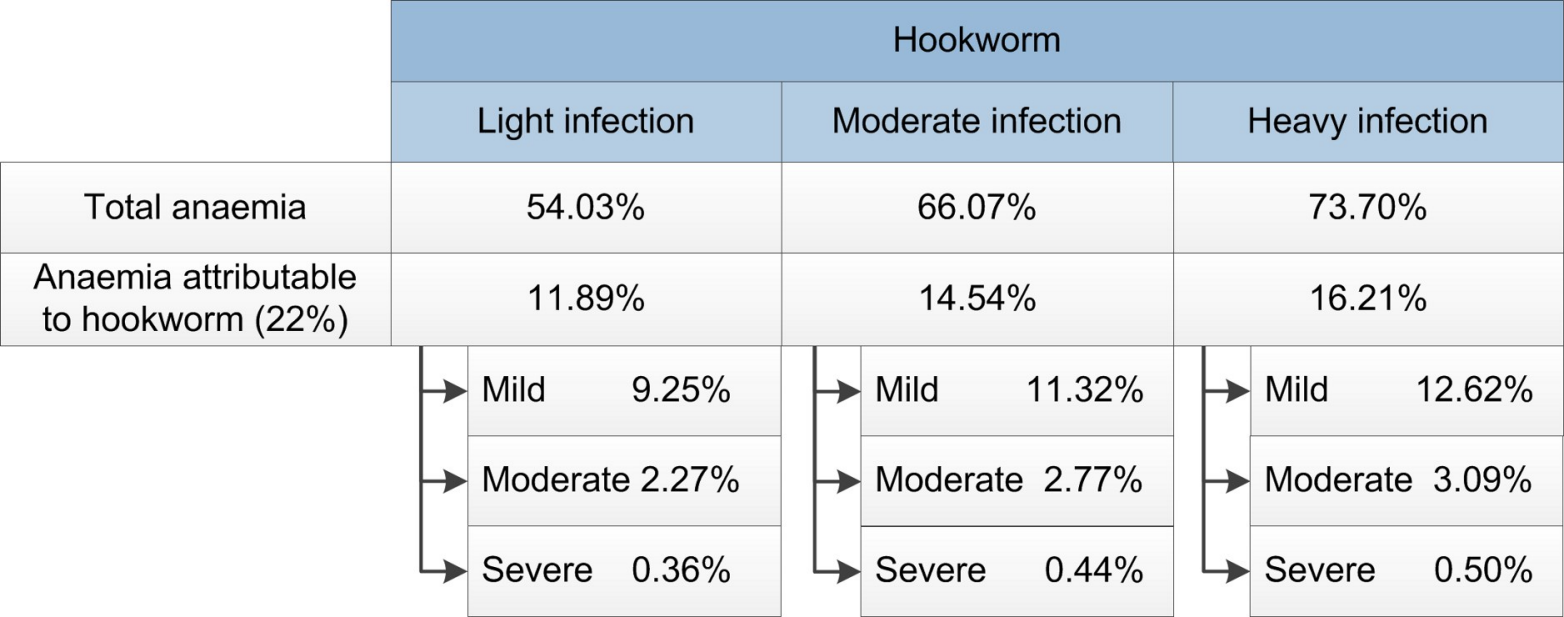
Prevalence of anaemia according to intensity of infection with hookworm.

The total anaemia prevalence and the anaemia attributable to hookworm were defined as shown in Fig 2, based on the weighted mean results of the studies detected in the literature review [33, 34, 37–40]. The anaemia prevalence was then subdivided into relative fractions of mild, moderate and severe anaemia: respectively 77.86%, 19.08% and 3.06% [41].

Mild and severe wasting are defined as having a weight-for-height score respectively below -2 and -3 standard deviations from the median of a reference population [42–44]. Only severe wasting was considered for the calculation of disability [45]. Based on input from six studies from a second literature review, we split up the total prevalence of wasting into relative fractions of mild wasting (65.75%) and severe wasting (34.25%) [46–51].

The total prevalence of wasting was obtained from the Vietnamese review of nutrition status in 2009–2010 [52]. We adopted the wasting prevalence for children under five years of age, which was reported as 7.1%. When multiplied with 34.25%, this results in an absolute prevalence of severe wasting of 2.43%. The results of the literature review can be found in Figure D in S1 Text.

Like anaemia, wasting is also caused by multiple factors. Many families in developing countries are affected by the poverty syndrome, a vicious cycle where low income, a large family size and poor education aggravate malnutrition over generations [53–55]. We arbitrarily defined the percentage of wasting improvable by deworming as 50%, leading to an absolute prevalence of severe wasting improvable by deworming of 1.22%. This was included in the sensitivity analysis (relatively ±50%) and proved not to be a sensitive parameter. Our assumption that deworming reduces wasting prevalence was supported by the literature [56–58].

The causes of disability for STH are linked to the associated disability weights from the most recent GBD update in 2013 and are outlined in Table 5 [45].

**Table 5:** Causes of disability and disability weights from the GBD 2013 associated with each intensity of STH infection [4, 45].

	Cause of disability	STH Intensity	Prevalence improvable by deworming (%)	Disability weight
**Hookworm**	Mild anaemia	All	Mild hookworm: 9.25 Moderate hookworm: 11.32 Heavy hookworm: 12.62	0.004
	Moderate anaemia	All	Mild hookworm: 2.27 Moderate hookworm: 2.77 Heavy hookworm: 3.09	0.052
	Severe anaemia	All	Mild hookworm: 0.36 Moderate hookworm: 0.44 Heavy hookworm: 0.50	0.149
	Mild abdominal problems	Moderate	100.00	0.011
	Severe wasting	Heavy	1.22	0.128
***A*. *lumbricoides***	Mild abdominal problems	Moderate	100.00	0.011
	Symptomatic infection	Heavy	100.00	0.027
	Severe wasting	Heavy	1.22	0.128
***T*. *trichiura***	Mild abdominal problems	Moderate	100.00	0.011
	Symptomatic infection	Heavy	100.00	0.027
	Severe wasting	Heavy	1.22	0.128

The DALYs were calculated separately for each worm type, differentiating between the three classes of intensity, according to the following formula:

$${\mathrm{DALY}}_{\left(i,t\right)}={\mathrm{DW}}_{\left(i\right)}\times p_{\left(i\right)}\times n_{\left(i,t\right)}$$

With DW_(i)_ the disability weight of disability cause i, weighted by the prevalence of that cause p_(i)_ and multiplied with the number of children having an infection intensity susceptible for that cause i at year t (n_(i,t)_) [27].

No age-weighting or discounting of disability were applied. When a person suffers from multiple disabilities a co-morbidity adjustment was applied, e.g. in the case of moderate hookworm infection 100% of patients suffer from mild abdominopelvic problems while 14.54% also suffer from different intensities of anaemia [27]. For each worm type and infection intensity, the average disability was calculated according to the following equation:

$${\mathrm{DW}}_{\left(1+2\right)}=1-\left(1-{\mathrm{DW}}_{\left(1\right)}\right)\times \left(1-{\mathrm{DW}}_{\left(2\right)}\right)$$

#### Assumptions and limitations

The prevalence of wasting was based on children under five years of age, who are the most vulnerable group. On the one hand, this might have been an overestimation because children aged 5–14 are less vulnerable to wasting. On the other hand, this might have been an underestimation because the wasting prevalence for individuals who are heavily infected with STH was now based on a sample from the general population, which diluted the effect. This was tested in the sensitivity analysis. The model calculations assumed an instantaneous recovery of disability when children were free of infection, while in practice this might take longer. There was a possibility of double counting when a person was infected by multiple worms. In that case the disability of wasting, symptomatic infection or abdominopelvic problems could have been counted multiple times, which is a limitation of the non-individualised cohort state-transition Markov model type [59]. A reduction of mortality associated with STH after deworming was not taken into account, which was considered a conservative approach [56, 58, 60]. We did not account for mental disability such as reduced cognitive development and school performance of children. We also did not consider the influence of initiatives other than deworming, such as Water, Sanitation and Hygiene (WASH) programs. The variation in coverage of anthelmintic treatment in Vietnam over the years was not taken into account in this simulation. We assumed a constant coverage of 80%, which is the same as the coverage that was reached in the program on which the Markov model (TPMS 4) was based (A. Montresor, personal communication).

### Part II: Public health burden

The public health burden of mebendazole Mass Drug Administration (MDA) was analysed through Life Cycle Assessment (LCA) methodology, which considered the full cradle-to-grave impact of the pharmaceutical supply chain. The resource use and emissions associated with the production of the medicine are linked to environmental cause-effect chains, enabling the quantification of the environmental impact [61–63]. For example, burning natural gas leads to an emission of CO_2_ (kg), which is associated with a radiative forcing in the atmosphere (W/m^2^), causing a global mean temperature increase (°C). The latter may lead to disability through direct effects of (extreme) heat or cold, foodborne and waterborne diseases, vector-borne diseases, natural disasters and risk of malnutrition [64, 65]. In this study we focus on the environmental impact which causes a global Human Health burden [17, 66].

#### Applied functional unit

The applied functional unit was the treatment with mebendazole 500 mg tablets every six months for five years of eight million children in Vietnam, aged 5–14, reaching 80% coverage [9]. In total 64 million tablets were required for this.

#### Goal and scope of the LCA

The study aimed to quantify the public health burden in Disability-Adjusted Life Years (DALY) associated with the pharmaceutical supply chain of mebendazole. The scope included the chemical synthesis of the Active Pharmaceutical Ingredient (API), tablet formulation, packaging, distribution and End-of-Life disposal. Resource use and emissions associated with health care utilization outside of the MDA program, e.g. hospitalization, was not taken into account.

#### Methods

The data were collected, processed and results provided according to the ISO 14040 and ISO 14044 standards and International Reference Life Cycle Data System (ILCD) guidelines [62, 63, 67, 68]. One exception was made regarding the use of Endpoint methods for the reporting of results.

#### Life cycle inventory

To feed the model, the inventory of the pharmaceutical supply chain was gathered from Janssen Pharmaceutica, and their external supply partners where primary data were necessary. The system boundaries were the limits of the production plants, however transport of intermediate products between plants was included, as well as the final transport to the children in Vietnam. We included the industrial unit operations as well as the plant supporting processes. We used primary data and the electricity mix was adapted concerning the origin of the electricity generation per production site.

The ecoinvent v3.1 database provided background data for the basic resources, e.g. energy and chemicals, entering the production system and waste treatment, extracted through SimaPro v8 software.

For the API synthesis, both data from the older production process of Janssen Pharmaceutica as from the current external supplier were used. Original Batch Production Records (BPRs) from the older process served as an input for the shortcut LCA Tool developed by Van der Vorst et al. [69], which were then updated with current process yields, provided by the external supplier. The resource use and associated emissions of the Heating, Ventilation and Air Conditioning (HVAC) system, not included in the shortcut LCA Tool, were collected from the external supplier.

Data for the tablet formulation and packaging was retrieved from Batch Production Reports, Cleaning Procedures, Equipment Manuals, MSDS files, yearly planning and P&ID’s from the external supplier. The indirect resource use of supporting processes such as HVAC, heating, cooling, generation of purified water and steam was included, together with waste treatment operations.

The distribution included the transport by truck and barge from the external supplier to Hanoi (Vietnam). From there we included car transport to distribute the mebendazole tablets to province and district level (D. Do Trung, personal communication). Distribution on community level was not included.

The End-of-Life phase considered the landfilling of the packaging waste, although in practice the empty bottles are frequently reused by the local population. The End-of-Life toxicity of mebendazole in continental freshwater was based on Environmental Risk Assessment (ERA) data such as the Bio Concentration Factor (BCF) and EC50 values. We excluded the fraction of API metabolised in the patient (10%) and assumed no removal by Wastewater Treatment after excretion by the patient (Janssen Pharmaceutica, personal communication).

#### Life cycle impact assessment

The ReCiPe v1.11 impact assessment method was used to quantify the public health burden induced by six environmental cause-effect chains: Climate Change, Human Toxicity, Ionizing Radiation, Ozone Depletion, Particulate Matter Formation and Photochemical Oxidant Formation [65, 66, 70, 71]. The hierarchical perspective was chosen [72–74].

The End-of-Life impact of the molecule was calculated through USEtox [63, 75, 76]. Using data from the ERA documents, USEtox applies a cause-effect chain that quantifies the number of disease cases associated with the mass (kg) of mebendazole released into the wastewater, based on fate, exposure and effect factors. As mebendazole is not a carcinogen, the average DALYs associated with non-cancer diseases (2.7) is applied per disease case [71].

The results of the LCA were divided according to the following direct inputs of the pharmaceutical supply chain: water, nitrogen, chemicals (reagents and solvents), energy (natural gas, electricity), packaging materials, industrial waste treatment, transport (pharmaceutical supply chain, distribution) and End-of-Life toxicity. Industrial waste treatment concerned the treatment of waste from production sites, whereas End-of-Life toxicity represented impact from post-consumer waste.

#### Assumptions and limitations

We considered only the pharmaceutical supply chain of mebendazole as a source of public health burden. This implies that no other health care utilization, e.g. hospitalization, was considered for its public health burden originating from resource use and emissions. Returns and destructions of medication were not assessed.

## Results

The modelled public health outcomes of five years mebendazole MDA compared to the baseline are listed in Table 6. The DALYs for the *A*. *lumbricoides* and *T*. *trichiura* treatment groups decreased over five years, while the DALYs of the hookworm treatment group increased and the DALYs of the ‘no treatment’ group stayed constant over time. This way mebendazole MDA averted 119,088 DALYs (89.32% reduction) for *A*. *lumbricoides* and 10,575 DALYs (70.46% reduction) for *T*. *trichiura* compared to the ‘no treatment’ group. For hookworm, there was an increase in DALYs of 13,076 (55.02% increase), which is a counterintuitive result as mebendazole is known to have efficacy against hookworm in terms of egg reduction rate, and thus morbidity [25, 77–79]. In total 116,587 DALYs are averted for the three worms combined, which is a reduction of 67.74%. The treatment effect was the most pronounced in the first year. After an initial drop in disability the values stabilized over time because an equilibrium with reinfection was reached [26].

**Table 6:** Disability-Adjusted Life Years (DALYs) after the introduction of mebendazole MDA for eight million Vietnamese children. The ‘no treatment’ group has DALY values equal to the baseline for each year.

	hookworm		*A*. *lumbricoides*		*T*. *trichiura*		
	DALYs after MDA	DALYs averted per year	DALYs after MDA	DALYs averted per year	DALYs after MDA	DALYs averted per year	Total cumulative DALYs averted
Baseline	4,754		26,667		3,002		
Year 1	6,861	-2,108	4,236	22,431	884	2,118	22,440
Year 2	7,433	-2,679	2,811	23,856	888	2,114	45,731
Year 3	7,514	-2,761	2,464	24,202	888	2,114	69,287
Year 4	7,518	-2,765	2,378	24,289	888	2,114	92,925
Year 5	7,517	-2,763	2,356	24,311	888	2,114	116,587
Total		-13,076		119,088		10,575	116,587

**Fig 3:**
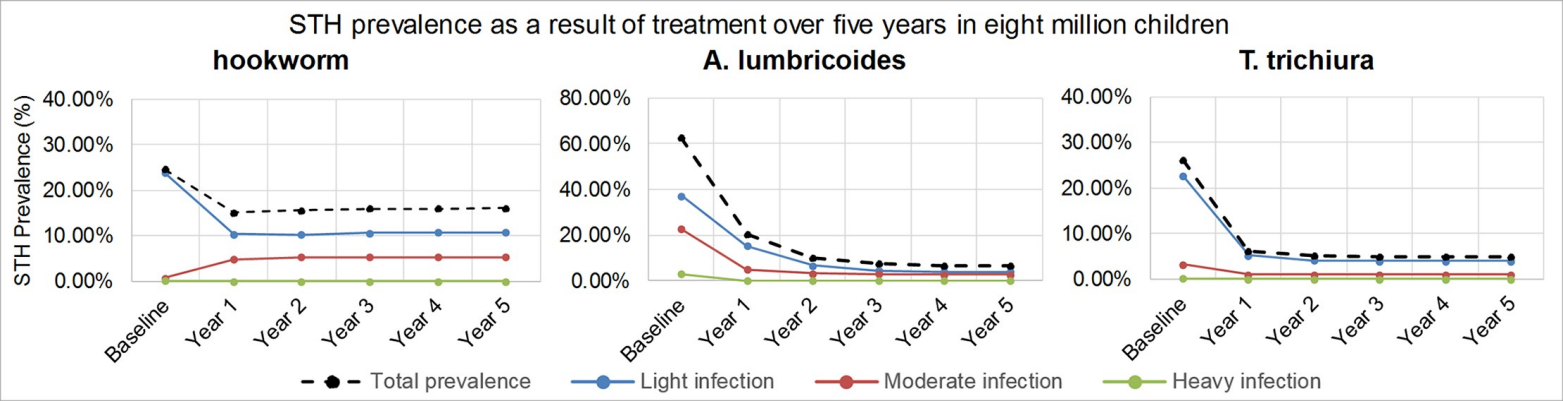
Soil-transmitted helminthiases (STH) prevalence over five years of mebendazole Mass Drug Administration (MDA) for eight million Vietnamese children.

**Fig 4:**
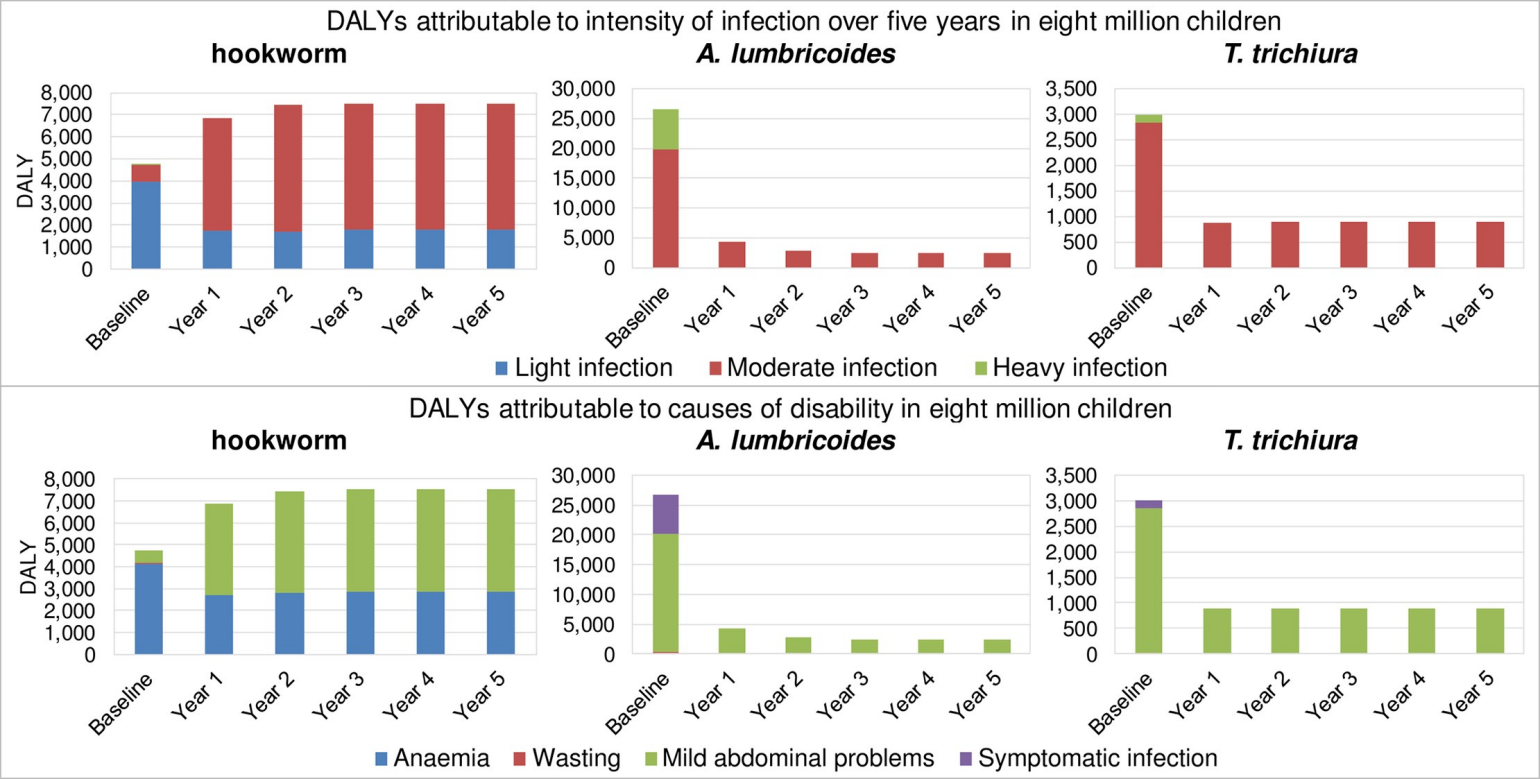
Soil-transmitted helminthiases (STH) DALYs attributable to both intensity of infection and cause of disability over five years of mebendazole Mass Drug Administration (MDA) for eight million Vietnamese children.

Fig 3 and Fig 4 provide more detail and display respectively the STH prevalence and DALYs attributable to both intensity of infection and cause of disability over five years of mebendazole MDA. Fig 3 shows that compared to the initial baseline values before treatment, the total prevalence absolutely decreased with 8.61% for hookworm, 56.18% for *A*. *lumbricoides* and 21.04% for *T*. *trichiura*. The prevalence of heavy infection in all three worms reached 0% after one year of treatment.

From the top part of Fig 4 it is notable that for *A*. *lumbricoides* and *T*. *trichiura* all DALYs are attributable to moderate and heavy infection at baseline, while after the first year of treatment all DALYs are attributable to moderate infection. For hookworm light infection is responsible for 83.79% of disability at baseline, while for later treatment years light infection and moderate infection are each responsible for around 25% and 75% of the DALYs, respectively. These trends can also be seen in the bottom part of Fig 4, where the DALYs attributable to mild abdominopelvic problems and symptomatic infection correspond with the public health burden of respectively moderate and heavy infection. For hookworm however, the largest share of DALYs at baseline can be attributed to anaemia, while after the first year of treatment mild abdominopelvic problems are responsible for the largest amount of DALYs.

At year 5, the fractions of DALYs averted from moderate infection compared to baseline were 88.15% and 68.77% for *A*. *lumbricoides* and *T*. *trichiura*, respectively. For hookworm, the DALYs from moderate infection increased with 687.2%. After the first year of treatment, 100% of the DALYs due to heavy infection are averted for all three worms.

The results of the scenario analysis on the percentage of anaemia attributable to hookworm are displayed in Table 7. In the most conservative scenario where 0% of anaemia is attributable to hookworm, the DALYs due to hookworm increased with 19,726 (negative DALYs averted) over five years of mebendazole MDA.

**Table 7:** Scenarios on the percentage of anaemia attributable to hookworm.

	Anaemia percentage attributable to hookworm	DALY averted for hookworm
Scenario 1: most conservative	0.00%	-19,726
Scenario 2: after introduction of MDA in Vietnam	4.12%	-18,481
Scenario 3: base case	22.00%	-13,076

The public health burden associated with the pharmaceutical supply chain is provided in Table 8. A public health burden of 6.46 DALYs was caused by the production, distribution and disposal of 64 million mebendazole tablets. API synthesis represented the largest impact (86.41%) with electricity and chemical reagents as the main drivers. Formulation of the tablets was responsible for the second largest impact, mainly due to electricity use. A table with results on midpoint impact categories can be found in Table E in S1 Text.

**Table 8:** Disability-Adjusted Life Years associated with the pharmaceutical supply chain of five years mebendazole MDA (64 million tablets).

	API Synthesis	Formulation	Packaging	Distribution	End-of-Life	Total
**Water**	0.002	0.000	0.000	-	-	0.002
**Nitrogen**	0.124	0.000	0.000	-	-	0.124
**Chemicals—Reagents**	1.846	0.033	0.000	-	-	1.880
**Chemicals—Solvents**	0.996	0.001	0.000	-	-	0.997
**Energy—Natural Gas**	0.058	0.002	0.000	-	-	0.060
**Energy—Electricity**	2.488	0.709	0.011	-	-	3.208
**Packaging materials**	0.000	0.000	0.042	-	-	0.042
**Industrial Waste Treatment**	0.036	0.012	0.000	-	-	0.049
**Transport—pharmaceutical supply chain**	0.036	0.000	0.000	-	-	0.100
**Transport—distribution to Vietnam**	-	-	-	0.064	-	0.004
**EOL Toxicity**	-	-	-	-	0.004	0.004
**Total**	5.586	0.757	0.053	0.064	0.004	6.465
	86.41%	11.72%	0.82%	0.99%	0.06%	

When comparing the public health benefit to the burden, even when including the theoretical increase in DALYs from hookworm infection, 18,035 times more DALYs were averted than created.

A one-way and probabilistic sensitivity analysis were carried out, to be found in Table F and Figure F, G, H and I in S1 Text. The conclusion did not change; in all cases the public health benefit because of mebendazole Mass Drug Administration was larger than the public health burden associated with the pharmaceutical supply chain. The starting prevalence for each worm was the most sensitive model parameter.

## Discussion

### Statement of principal findings

The treatment of eight million Vietnamese children with mebendazole for five years substantially decreased STH prevalence and averted 116,587 DALYs for the children compared to a ‘no treatment’ group, which is a reduction of 67,74%. To do this, 64 million mebendazole tablets are required, creating 6.46 DALYs associated with the pharmaceutical supply chain. A factor 18,035 more DALYs were averted than created. The increase in DALYs from hookworm infection is considered a counterintuitive result.

### Strengths and weaknesses of the study

This is one of the first attempts to compare the public health benefit of health care MDA programs to the public health burden associated with resource use and emissions of the pharmaceutical supply chain, using a common metric.

From an LCA perspective, there is a growing interest to quantify the benefit or handprint of products, rather than focus only on the environmental burdens [80, 81]. Simultaneously there is a willingness to include environmental assessments in health care decision making. A recent survey indicates that 71% of health care decision makers think the criteria of environmental impact should be considered when making decisions on health care interventions [82]. These developments suggest that there is support for a more holistic approach of health care interventions, which we aimed for in this study.

We aimed towards a transparent calculation of DALYs, using a previously published Markov model. The model was then linked with literature on the disability associated with STH.

The cradle-to-grave scope of the Life Cycle Assessment included the full pharmaceutical supply chain. The public health burden was based on multiple primary data sources. This holistic approach, combining different fields of research, could allow program managers to estimate the net public health performance of MDA programs.

The following limitations of the study should be noted. The modelling approach that was taken in this study was based on imperfect information. We aimed to provide the data inputs for the model through literature review, but given the paucity of certain data regarding STH some inputs were adopted from sources or settings that differ from the one in this study. For example, the Markov model transition probabilities were based on a study in Pemba island, Tanzania, instead of Vietnam. A full overview of model inputs, assumptions and limitations can be found in Table B, C and D in S1 Text.

The transition probabilities of the Markov model adopted in this study cause the prevalence of light, moderate and heavy infection to reach steady state values over five years treatment, regardless of the initial setting, i.e. starting prevalence [83]. In the case of moderate hookworm infection the starting prevalence (0.67%) is lower than the steady state prevalence (5.28%), causing a theoretical increase in both prevalence and DALYs, rather than a decrease. We consider this a counterintuitive result and the main limitation of the Markov model: it reports a decrease in health status, rather than an increase, when the starting prevalence is lower than the steady state value reached over time.

The relatively high steady state value after treatment for moderate hookworm may have been influenced by the high untreated hookworm prevalence in the population on which the TPMS 4 transition probabilities were based. In that study, the initial prevalence of moderate hookworm was 20.02%, with a total hookworm prevalence of 70.02% in 1324 children, which is 45.42% higher than the total hookworm prevalence in this study (A. Montresor, personal communication). These findings were also confirmed in a 1994 study in 3595 children from the same Pemba island, Tanzania, which reported moderate hookworm infection at 13.30%, with a total hookworm prevalence of 93.73% [37]. For *A*. *lumbricoides* and *T*. *trichiura* the initial prevalence in the population on which the model was based was 75.00 and 26.96%, respectively. These values are respectively 12.40 and 0.96% higher than the initial prevalence from Vietnam used in this study. It should also be noted that the source for STH prevalence in this study is an average of multiple provinces in Vietnam, which ignores the high variability across regions.

From the one-way and probabilistic sensitivity analysis in S1 Text it can be seen that a 50% relative change in starting prevalence can have a marked influence on the results. A what-if analysis showed that, all other values held constant, increasing the starting prevalence of moderate hookworm from 0.67% to 3.15% causes the total DALYs averted for hookworm to become 0. Further increasing said starting prevalence leads to positive DALYs averted. If the initial untreated prevalence is lower than the steady state values, it could be argued to keep the values constant from a model perspective, rather than let them increase. However, for the sake of transparency, we did not adapt the model. Another peculiar part of the transition matrix is the fact that for all three worms the probability of going from heavy infection to no infection is 1, instead of the more gradual decrease from heavy to moderate and then light infection that could be expected.

Aside from model considerations, the efficacy of mebendazole to treat hookworm has been reported as highly variable by Keiser et al. (2008) and more recently by Moser et al. (2017). As a result, combining hookworm data from different sources can possibly lead to higher prevalence after treatment compared to the baseline. Considering the limitations of the model, the results for hookworm are unlikely and should be conservatively interpreted.

The counterintuitive result for hookworm may lead to certain skepticism regarding the results for *A*. *lumbricoides* and *T*. *trichiura*. In this case, the cause for said counterintuitive result is a methodological weakness of the Markov model concept. The transition probabilities of a Markov model that predicts a treatment effect are always influenced by the pre-treatment situation, in this case the prevalence. The Markov model we used in this study was based on children in Tanzania, and while the starting prevalence for *A*. *lumbricoides* and *T*. *trichiura* in Vietnam were in line with the values from Tanzania, as discussed earlier, those for hookworm were not. Therefore the results for hookworm should be interpreted conservatively, but that does not follow for *A*. *lumbricoides* and *T*. *trichiura*.

We applied a static transmission model (constant probabilities), which has limitations compared to dynamic transmission modelling when considering communicable diseases [84]. While the linear transition probabilities in the Markov model allowed the prevalence of STH to reach a steady state value after year 2, MDA may actually lead to a complete elimination of the disease, which could be captured with the non-linear structure of a dynamic transmission model. Dynamic models may also include the indirect effects of treatment that arise from averted infections: while this study focuses on the treatment of children aged 5–14, it is possible that as a result younger children or adults have a reduced risk of infection because a lower fraction of the population is infected. Because of that, our results may be an underestimation of the treatment effect. Considering the specific case of comparing the public health benefit and burden of MDA programs, our findings clearly indicate that the benefit outweighs the burden. In that regard, applying a dynamic model may not be necessary [84]. An exception is probably the specific case of hookworm, where the quantification of DALYs over time may have benefited from a dynamic transmission model.

We aimed to use data from populations of Vietnamese children as much as possible. However, available published data was limited and in particular for the transition probabilities and assumptions regarding the prevalence of anaemia, data input originated from studies outside Vietnam: Brazil, Tanzania, Zimbabwe and Malaysia. There exist significant regional differences in the prevalence of anaemia, and although a general trend was observed across studies, Vietnamese data could have increased the validity of the outcomes.

The Life Cycle Assessment excluded any health care related resource use or emissions outside the pharmaceutical supply chain, e.g. the treatment of any co-morbidities in hospitals. Mebendazole MDA might increase the overall health of children, reducing the need for hospitalization and its associated resource use. However, the direct link between STH infection and hospitalization is not clear. This potential consequential reduction in environmental impact was not taken into account, which is considered conservative. There is a real possibility that a fraction of the tablets are lost before administration to the children in Vietnam (Janssen Pharmaceutica, personal communication). These losses were not taken into account, but would probably not have changed the main conclusions. Multiple anthelmintic drugs are donated for the treatment of STH, but data from the pharmaceutical supply chain was only collected for mebendazole. Because of that, other anthelmintic drugs such as Albendazole were not considered in this simulation.

In Life Cycle Impact Assessment multiple methods exist [63]. We adopted the grouping of environmental midpoint (effect) categories to calculate results on endpoint (damage) given the opportunity to directly compare the public health benefit and burden of MDA programs [85].

### Strengths and weaknesses in relation to other studies

A recent study by Montresor et al. quantified the DALYs that were averted from 2010 to 2015 by anthelmintic treatment, compared to the baseline morbidity present in 2010 due to the lack of large-scale treatment [86]. Similar to this study, the calculation of the averted DALYs is also based on the reduction in STH prevalence after treatment. The varying national coverage over the years was taken into account, contrary to the assumption in this study that the coverage remains constant. However, a more linear approach is used, linking the reduction in prevalence to a reduced morbidity through the elimination of cases of moderate and heavy intensity of infection, as defined by Marocco et al. [87]. The averted DALYs are quantified from a top-down approach adopting data from the WHO, rather than quantifying them separately for each intensity of infection [88]. The estimated fraction of averted DALYs for children aged 5–14 in the South East Asia Region is 64%, including widely varying treatment coverages. For the same age group and region, Montresor et al. estimate that 84% of DALYs could be averted by 2020 if 75% coverage is reached. The estimations in our study are more conservative and are more in line with the general South East Asia Region. The fact that hookworm theoretically increases DALYs instead of averting them in this study adds to the conservative nature of our estimate.

We compared the estimated DALYs from the Global Burden of Disease (GBD) report due to STH in Vietnam for children aged 5–14 with the results of this study. Although it is unclear to which degree the baseline STH prevalence in Vietnam of the GBD study compares to that of our study, the results from the GBD in 2005 and 2010 are generally consistent with our Markov simulation from 2006 to 2011 for *A*. *lumbricoides* and *T*. *trichiura*. For hookworm the large decrease in DALYs from the GBD is not visible in our study, as discussed earlier.

We identified one prior study that reports both patient outcomes and environmental impact for an, albeit different, pharmaceutical treatment [89]. However, as patient benefit and environmental burden are reported in two different metrics, the results of that study are not directly comparable to the outcomes of this study.

The literature retrieved in this study supports the claim that hookworm infection was associated with higher anaemia prevalence. We assumed the anaemia prevalence would then decrease with deworming. However, this was not confirmed by all reviews identified in the literature. While Gulani et al. and Smith et al. report increased haemoglobin after deworming, Hall et al. reported no effect. Taylor-Robinson et al., state that there was insufficient evidence to know whether deworming effects haemoglobin, although their approach diverges from the other reviews in that mainly the effect of MDA on an unscreened population was quantified. Due to the high number of individuals that are either not infected or have a light infection, the treatment effect on the smaller fraction of moderate and heavily infected individuals may have been diluted [30, 36, 57, 58, 90–92]. As shown in the scenario analysis, the conclusion of the study holds even with no reduction of anaemia prevalence due to treatment.

For wasting there is more agreement that deworming increases weight and height, supporting the assumptions of this study. Hall et al. state that deworming leads to significant extra gains in weight and height if STH prevalence is above 50% [90]. Taylor-Robinson et al. report that treating infected children with a single deworming dose may increase weight gain over the next six months [57]. While Welch et al. note little to no improvement on weight or height 12 months after mass deworming, a subgroup analysis on children with STH prevalence >20% does suggest weight gain [56].

### Implications of the study

The results of this study suggest that MDA of mebendazole for the treatment of STH substantially averts disability, based on limited evidence for children aged 5–14 in Vietnam, even if the public health burden of the pharmaceutical supply chain is fully considered. The public health burden associated with the pharmaceutical supply chain of mebendazole is negligible when compared to the public health benefit and expressed in a common metric.

This methodology may be useful if future policy would place a heavy emphasis on environmental considerations. For example, we could consider a future where the effectiveness of the treatment would not only be compared to the monetary costs (cost-effectiveness) but also to the environmental impact. Furthermore, consider a scenario where the limiting factor of the total national health care budget is accompanied with a limited total environmental impact e.g. through managed decline of emissions [93]. Next to cost-effectiveness, this would require a framework to simultaneously evaluate the environmental burden and the effectiveness of pharmaceutical treatments. We aimed to propose such a framework in the current study.

### Unanswered questions and future research

The main limitation of this study is related to the transition probabilities of the Markov model, which reach a steady state value after several years of treatment, allowing DALYs to increase if the initial prevalence is below the steady state value, as is the case with hookworm. While the Markov model has advantages with respect to transparency and reproducibility, the latter issue could be addressed in future research by developing transition probabilities that adapt to the starting prevalence. A future study with a singular focus on the treatment benefit over time (and not including the burden through Life Cycle Assessment) may apply a dynamic transmission model to address the same previously mentioned issue. Quantitative evidence on the long-term influence of STH infection on developmental and cognitive abilities is required to include this disability in future models. The influence of treatment of co-morbidities, both for the patient and the environment, should be included to capture the broader public health impact of STH infection. The influence of Water, Sanitation and Hygiene (WASH) programs on the health of children infected with STH could be included in future studies next to deworming. Health care utilization other than deworming, e.g. hospitalization, could be included if there is a clear link with STH infection.

The results of this study and their generalizability should be validated with research on other disease areas, countries, health care settings and standards. The environmental part of this study is limited to impacts on public health. Other environmental Areas of Protection (AoP), such as depletion of natural resources and damage to ecosystems may be considered for inclusion in future studies to capture all environmental aspects.

We considered the specific case of mebendazole, and many challenges still remain to capture the full impact of STH infection. However, this study provides a first insight on the public health benefit and burden of MDA programs, evaluated with a holistic approach that includes both the treatment benefit for the patient and the public health burden induced by the pharmaceutical supply chain. Such a methodology might be useful for policymakers interested in a holistic approach towards the two scientific fields that are described.

## Supporting information

S1 Text Supporting Information on the model choice, inputs, assumptions, literature reviews, sensitivity analysis and alternative Life Cycle Assessment reporting by midpoint impact categories. (DOCX)
