# Supplementary material for: The public health benefit and burden of mass drug administration programs in Vietnamese schoolchildren: Impact of mebendazole
Source: PLoS Negl Trop Dis. 2018 Nov 12;12(11):e0006954. doi: 10.1371/journal.pntd.0006954 (PMC6258429; doi:10.1371/journal.pntd.0006954)
Supplement: S1 Text — (DOCX) [file pntd.0006954.s001.docx]

**Supporting Information**

The public health benefit and burden of mass donation programs in Vietnamese schoolchildren: impact of mebendazole

Sam Debaveye^a^, Claudia Virginia Gonzalez Torres^a^, Delphine De Smedt^b^, Bert Heirman^c^, Shane Kavanagh^d^, Jo Dewulf^a^

*^a^ Research Group Environmental Organic Chemistry and Technology (EnVOC), Faculty of Bioscience Engineering, Ghent University, Campus Coupure, Coupure Links 653, B-9000 Ghent, Belgium*

*^b^ Department of Public Health, Ghent University, Campus UZ, De Pintelaan 185, B-9000, Ghent, Belgium*

*^c^ Johnson & Johnson EHS&S, Janssen Pharmaceutica NV, Turnhoutseweg 30, B-2340 Beerse, Belgium*

*^d^ Health Economics, Janssen Pharmaceutica NV, Turnhoutseweg 30, B-2340 Beerse, Belgium*

Corresponding author:

Sam Debaveye

Sam.Debaveye@UGent.be

T +32 9 264 59 48

F +32 9 264 62 43

Research Group Environmental Organic Chemistry and Technology (EnVOC), Faculty of Bioscience Engineering, Ghent University, Coupure Links 653, Ghent B-9000, Belgium

# Model choice

In this study we aimed to model the health effects of pharmaceutical treatment with mebendazole every six months to treat soil-transmitted helminthiases (STH) in children aged 5-14 in Vietnam. Multiple model types are available in the literature that represent STH disease progression [[1-5](#_ENREF_1)]. We selected the Markov model type with models published by A. Montresor, as they can be applied to hookworm, *A. lumbricoides* and *T. trichiura*, are split up in four classes of infection (no infection, light infection, moderate infection and heavy infection), based on eggs per gram (epg), and can be directly linked to disability weights in a transparent and reproducible way [[6](#_ENREF_6)].

The main model published by [Montresor et al. (2013)](#_ENREF_6) is based on 178 Vietnamese adult women that were treated with albendazole every six months [[6](#_ENREF_6)]. Our study aimed to model the effect of a different medicine on another age group: mebendazole on school-aged children. We considered the additional models published by [Montresor et al. (2016)](#_ENREF_7), which provide Transition Probability Matrix Sets (TPMS) for ten different interventions [[7](#_ENREF_7)]. As we aimed to model treatment with mebendazole every six months, TPMS 2 seems the preferred choice as it is based on said treatment of 1311 school-aged children in Tanzania. However, the transition probabilities do not always sum exactly to 1 in each row, which is a prerequisite for a sound Markov model. Therefore we chose to adopt TPMS 4, which represents treatment with mebendazole once per year, and is based on 1324 school-aged children in Tanzania. The treatment of children once per year instead of twice per year is a limitation, as World Health Organization (WHO) guidelines recommend six-monthly treatment if the STH prevalence is higher than 50% [[8](#_ENREF_8)]. However we would expect the results to be conservative. The fact that the model is based on Tanzania instead of Vietnam is a limitation.

Table A: Transition Probability Matrix Set 4 [[7](#_ENREF_7)].

| **hookworm** | | | | |
| --- | --- | --- | --- | --- |
|  | **Non infected** | **Light infection** | **Medium infection** | **High infection** |
| Non infected | 0.9010 | 0.0440 | 0.0550 | 0.0000 |
| Light infection | 0.7028 | 0.2752 | 0.0220 | 0.0000 |
| Medium infection | 0.1500 | 0.7700 | 0.0800 | 0.0000 |
| High infection | 1.0000 | 0.0000 | 0.0000 | 0.0000 |
| ***T. trichiura*** | | | | |
|  | **Non infected** | **Light infection** | **Medium infection** | **High infection** |
| Non infected | 0.9535 | 0.0364 | 0.0101 | 0.0000 |
| Light infection | 0.8989 | 0.0910 | 0.0101 | 0.0000 |
| Medium infection | 0.8633 | 0.1280 | 0.0088 | 0.0000 |
| High infection | 1.0000 | 0.0000 | 0.0000 | 0.0000 |
| ***A. lumbricoides*** | | | | |
|  | **Non infected** | **Light infection** | **Medium infection** | **High infection** |
| Non infected | 0.9530 | 0.0230 | 0.0240 | 0.0000 |
| Light infection | 0.7100 | 0.2300 | 0.0600 | 0.0000 |
| Medium infection | 0.6650 | 0.2600 | 0.0750 | 0.0000 |
| High infection | 1.0000 | 0.0000 | 0.0000 | 0.0000 |

# Overview of model inputs and assumptions

Table B: Overview of Markov model inputs.

| Model input | Source |
| --- | --- |
| Model source (TPMS 4 in Supporting Information) | [Montresor et al. (2016)](#_ENREF_7) |
| STH starting prevalence in untreated population of Vietnamese children aged 5-15 | [van der Hoek et al. (2003)](#_ENREF_9) |
| Distribution of total prevalence in light, moderate and heavy infection | [Montresor et al. (2014)](#_ENREF_10) |
| Disability causes of STH | [Pullan et al. (2014)](#_ENREF_11) |
| Disability values of STH | [Salomon et al. (2015)](#_ENREF_12) |
| Prevalence of anaemia in hookworm-infected children (weighted average of five studies) | [Ngui et al. (2012)](#_ENREF_13), [Midzi et al. (2010)](#_ENREF_14), [Brooker et al. (2007)](#_ENREF_15), [Guyatt et al. (2001)](#_ENREF_16), [Stoltzfus et al. (1997)](#_ENREF_17) |
| Distribution of anaemia in mild, moderate and severe anaemia | [Pasricha et al. (2008)](#_ENREF_18) |
| Fraction of anaemia attributable to hookworm: base case | [Nguyen et al. (2006)](#_ENREF_19) |
| Fraction of anaemia attributable to hookworm: scenario 2 after introduction of MDA in Vietnam | [Nguyen et al. (2015)](#_ENREF_20) |
| Prevalence of wasting | [National Institute of Nutrition (2011)](#_ENREF_21) |
| Distribution of total wasting prevalence into mild and severe wasting (average of six studies) | [*Munisi et al. (2016)*](#_ENREF_22)*,* [*Salim et al. (2014)*](#_ENREF_23)*,* [*Winkler et al. (2014)*](#_ENREF_24)*,* [*Bustinduy et al. (2013)*](#_ENREF_25)*,* [*Casapía et al. (2007)*](#_ENREF_26)*,* [*Fernando et al. (2000)*](#_ENREF_27) |
| Calculation of co-morbidity adjustment | [WHO (2017)](#_ENREF_28) |

Table C: Overview of main study assumptions.

| Assumptions | Implication |
| --- | --- |
| Fraction of wasting improvable by deworming: 50% | Unclear due to lack of data on real-world wasting improvable by deworming |
| Prevalence of wasting based on Vietnamese children <5 years of age | Rationale for underestimation: value based on general population of children instead of STH infected  Rationale for overestimation: wasting prevalence is typically highest in children <5 years of age |
| Instantaneous recovery of disability after moving to state with lower intensity of infection | Overestimation of DALYs averted |
| Worm prevalence and morbidity stay constant in the counterfactual ‘no treatment’ over time on a population level | Unclear due to lack of data |
| Medicine coverage of mebendazole remains constant at 80% | Markov model is applied identically each year |

Table D: Overview of main study limitations.

| Limitations | Implication |
| --- | --- |
| Double counting of disability if a person simultaneously suffers from multiple worms | Overestimation of DALYs averted |
| Mortality not taken into account | Results are conservative |
| Mental disability not taken into account | Results are conservative |
| Markov model: 1x mebendazole/year instead of 2x mebendazole/year | Underestimation of DALYs averted |
| Markov model: steady state value of moderate hookworm infection is higher than initial value | Underestimation of DALYs averted |

# Literature review to support prevalence of anaemia and wasting

**Search terms**:

(hookworm*[Title/Abstract] OR "hook worm*"[Title/Abstract] OR Ancylostomatoidea[mesh:noexp] OR "Ancylostoma duodenale"[Title/Abstract] OR Necator americanus[Title/Abstract] OR necator americanus[mesh] OR ancylostoma[mesh] OR hookworm infections[mesh] OR nematoda[mesh:noexp]

OR ascaris[Title/Abstract] OR ascaris[mesh] OR Ascaris lumbricoides[Title/Abstract] OR roundworm*[Title/Abstract] OR "round worm*"[Title/Abstract] OR ascaris lumbricoides[mesh] OR ascaris infections[mesh] OR ascariasis[mesh] OR ascariasis[Title/Abstract]

OR trichuris[Title/Abstract] OR "Trichuris trichiura"[Title/Abstract] OR whipworm[Title/Abstract] OR "whip worm*"[Title/Abstract] OR trichuris[mesh] OR trichuriasis[mesh])

AND (anaemi*[Title/Abstract] OR anemi*[Title/Abstract] OR anemia[mesh] OR wasting[Title/Abstract] OR "weight loss"[Title/Abstract] OR weight loss[mesh] OR "weight gain"[Title/Abstract] OR weight gain[mesh] OR wasting[mesh] OR wasting[Title/Abstract])

AND (prevalence[Title/Abstract] OR prevalence[mesh:noexp] OR Epidemiology[mesh:noexp])

AND (children[Title/Abstract] OR child[Title/Abstract] OR child[mesh] OR infant[Title/Abstract] OR infants[Title/Abstract] OR Baby[Title/Abstract] OR Babies[Title/Abstract] OR Toddler*[Title/Abstract] OR Preschool[Title/Abstract] OR Preschooler*[Title/Abstract] OR Preadolescent*[Title/Abstract] OR Pediatric[Title/Abstract] OR Paediatric[Title/Abstract])

**Databases**: Medline, Cochrane reviews, EMBASE, LILACS, WHOLIS, POPLINE

**Date searched**: 14/02/2017

**Dates searched**: 2000 – 2017

## Literature identified on anaemia prevalence for light, moderate and heavy hookworm

PRISMA diagram is displayed in Figure A.


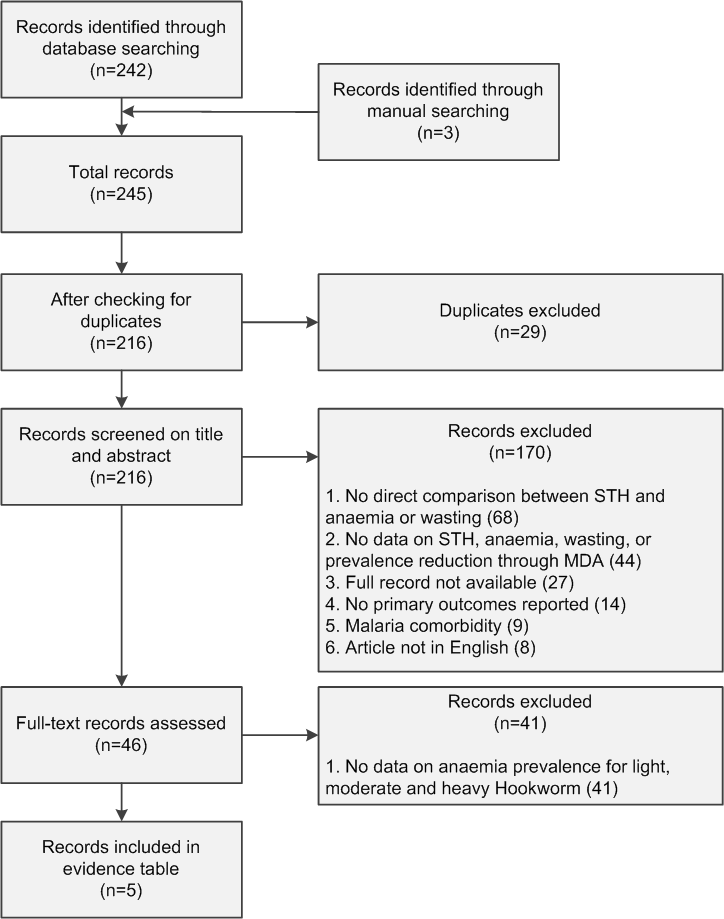


Figure A: PRISMA diagram of the literature review on the anaemia prevalence for light, moderate and heavy hookworm.

**Conclusion:** weighted average of five studies [[13-17](#_ENREF_13)], of which the results are graphically represented in Figure B.

Figure B: Prevalence of anaemia in pre-School Age Children (pre-SAC) and School Age Children (SAC) infected with hookworm.

## Literature identified on the anaemia subdivision in mild, moderate and severe anaemia

PRISMA diagram is displayed in Figure C.


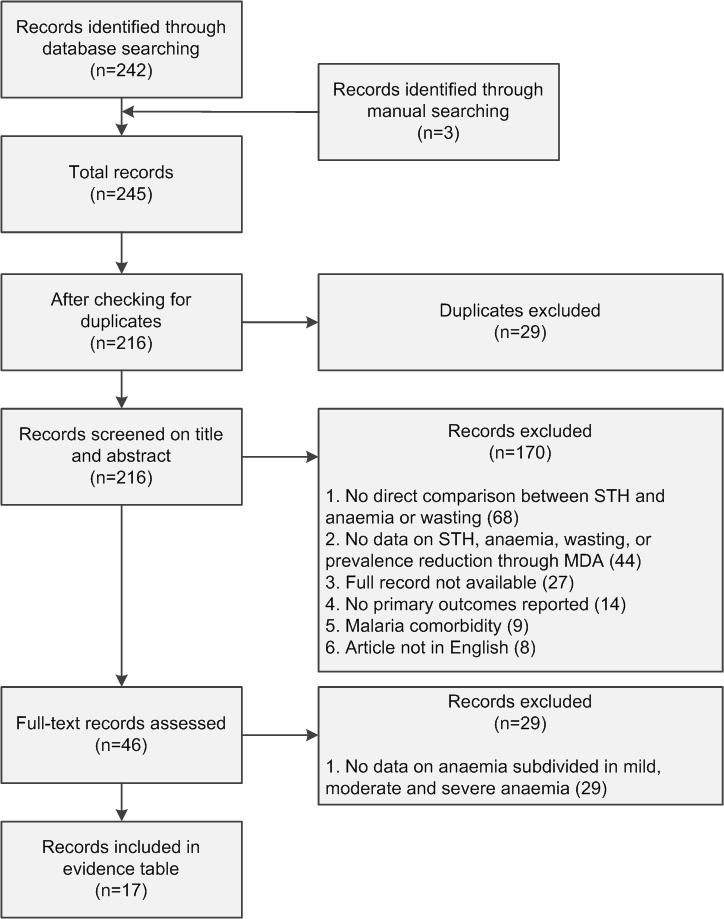


Figure C: PRISMA diagram of the literature review on the anaemia subdivision in mild, moderate and severe anaemia.

Conclusion: most conservative record is chosen out of evidence table [[18](#_ENREF_18)].

## Literature identified on the prevalence of mild and severe wasting

PRISMA diagram is displayed in Figure D.

###
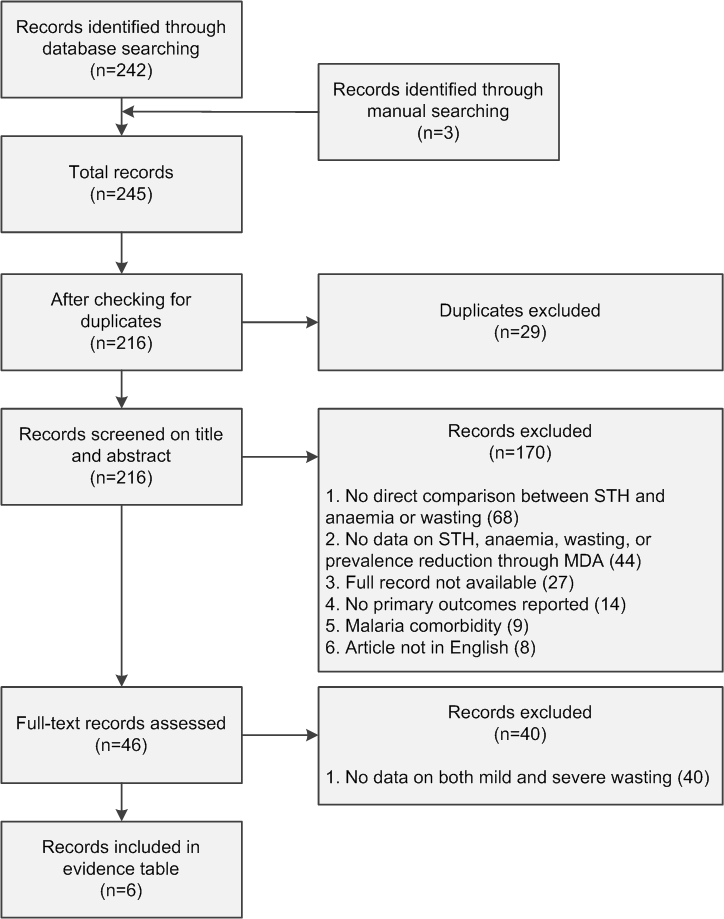


Figure D: PRISMA diagram of the literature review on the the prevalence of mild and severe wasting.

Conclusion: average of six studies [[22-27](#_ENREF_22)].

# Literature review to support the fraction of anaemia attributable to hookworm

**Search terms**:

(hookworm*[Title/Abstract] OR "hook worm*"[Title/Abstract] OR Ancylostomatoidea[mesh:noexp] OR "Ancylostoma duodenale"[Title/Abstract] OR Necator americanus[Title/Abstract] OR necator americanus[mesh] OR ancylostoma[mesh] OR hookworm infections[mesh] OR nematoda[mesh:noexp])

AND (anaemi*[Title/Abstract] OR anemi*[Title/Abstract] OR anemia[mesh])

AND (prevalence[Title/Abstract] OR prevalence[mesh:noexp] OR Epidemiology[mesh:noexp]) AND (uninfected[Title/Abstract] OR non-infected[Title/Abstract] OR "non infected"[Title/Abstract] OR "not infected"[Title/Abstract] OR "uninfested"[Title/Abstract] OR "non-infested"[Title/Abstract] OR "non infested"[Title/Abstract] OR "not infested"[Title/Abstract])

AND (children[Title/Abstract] OR child[Title/Abstract] OR child[mesh] OR infant[Title/Abstract] OR infants[Title/Abstract] OR Baby[Title/Abstract] OR Babies[Title/Abstract] OR Toddler*[Title/Abstract] OR Preschool[Title/Abstract] OR Preschooler*[Title/Abstract] OR Preadolescent*[Title/Abstract] OR Pediatric[Title/Abstract] OR Paediatric[Title/Abstract])

**Databases**: Medline, EMBASE, LILACS, WHOLIS, POPLINE

**Date searched**: 07/06/2017

**Dates searched**: 2000 – 2017

PRISMA diagram is displayed in Figure E.

###
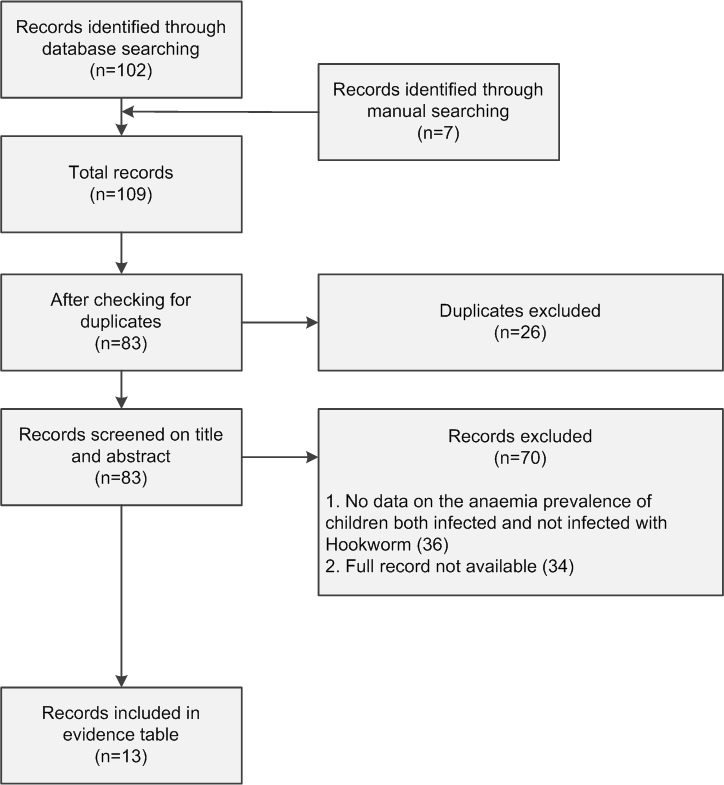


Figure E: PRISMA diagram of the literature review on the fraction of anaemia attributable to hookworm.

Conclusion: assumption that hookworm is associated with higher anaemia prevalence supported by evidence from 13 studies [[13-17](#_ENREF_13), [29-36](#_ENREF_29)].

# Life Cycle Impact Assessment

Results of the environmental Life Cycle Assessment with subdivision of midpoint impact categories.

Table E: Disability-Adjusted Life Years associated with the pharmaceutical supply chain of five years mebendazole MDA (64 million tablets).

|  | **API Synthesis** | **Formulation** | **Packaging** | **Distribution** | **End-of-Life** | **Total** |
| --- | --- | --- | --- | --- | --- | --- |
| **Climate Change** | 3.749 | 0.517 | 0.041 | 0.032 | 0.003 | 4.342 |
| **Human Toxicity** | 0.434 | 0.065 | 0.002 | 0.003 | 0.001 | 0.505 |
| **Ionising Radiation** | 0.004 | 0.001 | 0.000 | 0.000 | 0.000 | 0.005 |
| **Ozone Depletion** | 0.001 | 0.000 | 0.000 | 0.000 | 0.000 | 0.001 |
| **Particulate Matter Formation** | 1.398 | 0.175 | 0.010 | 0.028 | 0.000 | 1.611 |
| **Photochemical Oxidant Formation** | 0.000 | 0.000 | 0.000 | 0.000 | 0.000 | 0.000 |
|  |  |  |  |  |  |  |
| **Total** | 5.586 | 0.757 | 0.053 | 0.064 | 0.004 | 6.465 |
|  | 86.41% | 11.72% | 0.82% | 0.99% | 0.06% |  |

# One-way sensitivity analysis

A One-way Sensitivity Analysis was performed to check the robustness of the model with respect to the starting prevalence of light, moderate and heavy infection for each of the worms, the prevalence of anaemia and wasting and the fraction of anaemia and wasting that is improvable by deworming. The base case parameters were relatively varied ±50%, except for the percentage of anaemia improvable, where the minimum value was set to 0% and the maximum value to 150% of the base case value (22%): 33%. The value of ±50% was adopted to sufficiently test the sensitivity of the parameters as no probability distribution or standard deviation was provided along with the inputs of the model that are tested in the sensitivity analysis. Table F provides the min. and max. values to which the parameters were varied.

Table F: Parameters varied in the sensitivity analysis.

| Parameters | Min. value (%) | Base case value (%) | Max. value (%) |
| --- | --- | --- | --- |
| Start prevalence, hookworm, light | 11.90 | 23.81 | 35.71 |
| Start prevalence, hookworm, moderate | 0.34 | 0.67 | 1.01 |
| Start prevalence, hookworm, heavy | 0.06 | 0.12 | 0.18 |
| Start prevalence, *A. lumbricoides*, light | 18.52 | 37.03 | 55.55 |
| Start prevalence, *A. lumbricoides*, moderate | 11.30 | 22.59 | 33.89 |
| Start prevalence, *A. lumbricoides*, heavy | 1.49 | 2.97 | 4.46 |
| Start prevalence, *T. trichiura*, light | 11.35 | 22.70 | 34.05 |
| Start prevalence, *T. trichiura*, moderate | 1.61 | 3.23 | 4.84 |
| Start prevalence, *T. trichiura*, heavy | 0.04 | 0.07 | 0.11 |
| % anaemia improvable | 0.00 | 22.00 | 33.00 |
| Total anaemia prevalence, light hookworm | 27.01 | 54.03 | 81.04 |
| Total anaemia prevalence, Moderate hookworm | 33.04 | 66.07 | 99.11 |
| Total anaemia prevalence, heavy hookworm | 36.85 | 73.70 | 100 |
| Wasting prevalence | 1.22 | 2.43 | 3.65 |
| % wasting improvable | 25.00 | 50.00 | 75.00 |

Results are presented in Figure F, Figure G and Figure H as Disability-Adjusted Life Years (DALYs) avoided for 5 years of deworming. The percentage of anaemia improvable was the most sensitive parameter.

Figure F: Tornado diagram displaying the sensitivity of the model to key input parameters for hookworm.

Figure G: Tornado diagram displaying the sensitivity of the model to key input parameters for *A. lumbricoides*.

Figure H: Tornado diagram displaying the sensitivity of the model to key input parameters for *T. trichiura*.

# Probabilistic sensitivity analysis

The Probabilistic Sensitivity Analysis (PSA) presented in Figure I includes the same parameters as the One-way Sensitivity Analysis and randomly varies the parameters within the same lower and upper limits, using a continuous uniform distribution. The PSA ran for 10,000 iterations and the results are presented as avoided DALYs for hookworm, *A. Lumbricoides*, *T. Trichiura* and the sum total for the three worms combined. The boxplot displays the minimum, Q1, median, Q3 and maximum values.


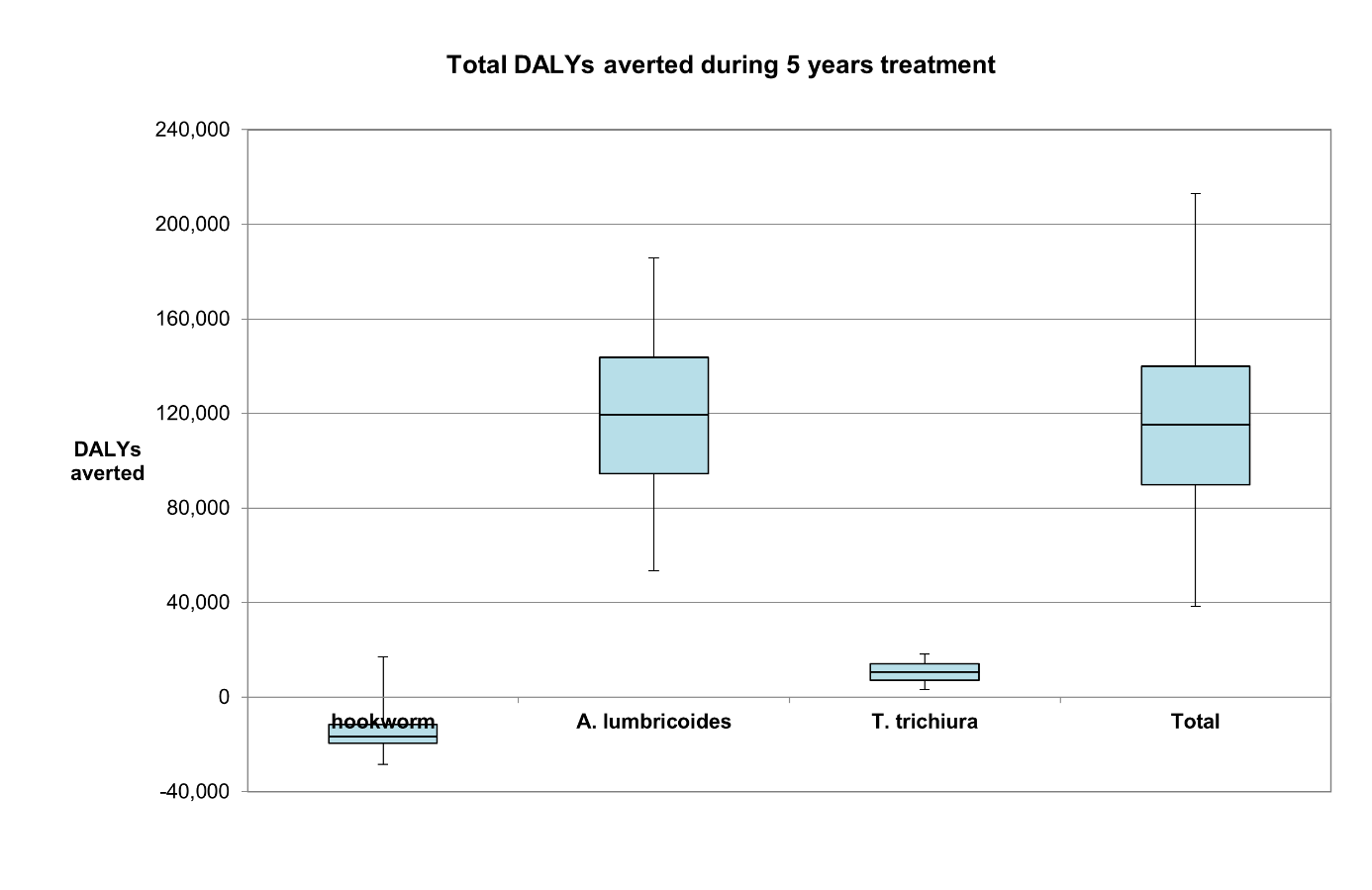


Figure I: Results of the Probabilistic Sensitivity Analysis displayed as a boxplot.

# References

1. Coffeng LE, Bakker R, Montresor A, de Vlas SJ. Feasibility of controlling hookworm infection through preventive chemotherapy: a simulation study using the individual-based WORMSIM modelling framework. Parasites & Vectors. 2015;8(1):541. doi: 10.1186/s13071-015-1151-4.

2. Lwambo NJ, Bundy DA, Medley GF. A new approach to morbidity risk assessment in hookworm endemic communities. Epidemiol Infect. 1992;108(3):469-81. doi: <https://doi.org/10.1017/s0950268800049980>.

3. Truscott J, Hollingsworth TD, Anderson R. Modeling the Interruption of the Transmission of Soil-Transmitted Helminths by Repeated Mass Chemotherapy of School-Age Children. PLoS Negl Trop Dis. 2014;8(12):e3323. doi: 10.1371/journal.pntd.0003323. PubMed PMID: PMC4256169.

4. Truscott JE, Turner HC, Farrell SH, Anderson RM. Chapter Three - Soil-Transmitted Helminths: Mathematical Models of Transmission, the Impact of Mass Drug Administration and Transmission Elimination Criteria. In: Basáñez MG, Anderson RM, editors. Adv Parasitol. 94: Academic Press; 2016. p. 133-98.

5. Chan MS, Guyatt HL, Bundy DA, Medley GF. The development and validation of an age-structured model for the evaluation of disease control strategies for intestinal helminths. Parasitology. 1994;109. doi: 10.1017/s0031182000078422.

6. Montresor A, Gabrielli AF, Yajima A, Lethanh N, Biggs B, Casey GJ, et al. Markov model to forecast the change in prevalence of soil-transmitted helminths during a control programme: a case study in Vietnam. Trans R Soc Trop Med Hyg. 2013;107(5):313-8. doi: <https://doi.org/10.1093/trstmh/trt019>.

7. Montresor A, Deol A, à Porta N, Lethanh N, Jankovic D. Markov Model Predicts Changes in STH Prevalence during Control Activities Even with a Reduced Amount of Baseline Information. PLoS Negl Trop Dis. 2016;10(4):e0004371. doi: 10.1371/journal.pntd.0004371. PubMed PMID: PMC4817985.

8. WHO. Guideline: preventive chemotherapy to control soil-transmitted helminth infections in at-risk population groups. Geneva: World Health Organization, 2017.

9. van der Hoek W, De NV, Konradsen F, Cam PD, Hoa NTV, Toan ND, et al. Current status of soil-transmitted helminths in Vietnam. Southeast Asian J Trop Med Public Health. 2003;34 Suppl 1:1-11. PubMed PMID: 12971504.

10. Montresor A, à Porta N, Albonico M, Gabrielli AF, Jankovic D, Fitzpatrick C, et al. Soil-transmitted helminthiasis: the relationship between prevalence and classes of intensity of infection. Trans R Soc Trop Med Hyg. 2014;109(4):262-7. doi: <https://doi.org/10.1093/trstmh/tru180>.

11. Pullan RL, Smith JL, Jasrasaria R, Brooker SJ. Global numbers of infection and disease burden of soil transmitted helminth infections in 2010. Parasit Vectors. 2014;7(1):1-19. doi: <https://doi.org/10.1186/1756-3305-7-37>.

12. Salomon JA, Haagsma JA, Davis A, de Noordhout CM, Polinder S, Havelaar AH, et al. Disability weights for the Global Burden of Disease 2013 study. Lancet Glob Health. 2015;3(11):e712-e23. doi: <https://doi.org/10.1016/S2214-109X(15)00069-8>.

13. Ngui R, Lim YAL, Chong Kin L, Sek Chuen C, Jaffar S. Association between Anaemia, Iron Deficiency Anaemia, Neglected Parasitic Infections and Socioeconomic Factors in Rural Children of West Malaysia. PLoS Negl Trop Dis. 2012;6(3):e1550. doi: <https://doi.org/10.1371/journal.pntd.0001550>. PubMed PMID: PMC3295806.

14. Midzi N, Mtapuri-Zinyowera S, Mapingure MP, Sangweme D, Chirehwa MT, Brouwer KC, et al. Consequences of polyparasitism on anaemia among primary school children in Zimbabwe. Acta Trop. 2010;115(1):103-11. doi: <http://dx.doi.org/10.1016/j.actatropica.2010.02.010>.

15. Brooker S, Jardim-Botelho A, Quinnell RJ, Geiger SM, Caldas IR, Fleming F, et al. Age-related changes in hookworm infection, anaemia and iron deficiency in an area of high Necator americanus hookworm transmission in south-eastern Brazil. Trans R Soc Trop Med Hyg. 2007;101(2):146-54. doi: <https://doi.org/10.1016/j.trstmh.2006.05.012>.

16. Guyatt HL, Brooker S, Kihamia CM, Hall A, Bundy DA. Evaluation of efficacy of school-based anthelmintic treatments against anaemia in children in the United Republic of Tanzania. Bull World Health Organ. 2001;79(8):695-703. PubMed PMID: PMC2566500.

17. Stoltzfus RJ, Chwaya HM, Tielsch JM, Schulze KJ, Albonico M, Savioli L. Epidemiology of iron deficiency anemia in Zanzibari schoolchildren: the importance of hookworms. Am J Clin Nutr. 1997;65(1):153-9.

18. Pasricha S, Caruana SR, Phuc TQ, Casey GJ, Jolley DJ, Kingsland SR, et al. Anemia, iron deficiency, meat consumption, and hookworm infection in women of reproductive age in northwest Vietnam. Am J Trop Med Hyg. 2008;78(3):375-81.

19. Nguyen PH, Nguyen KC, Le MB, Nguyen TV, et al. Risk factors for anemia in Vietnam. Southeast Asian J Trop Med Public Health. 2006;37(6):1213-23. PubMed PMID: 201458169; 17333780.

20. Nguyen PH, Gonzalez-Casanova I, Nguyen H, Pham H, Truong TV, Nguyen S, et al. Multicausal etiology of anemia among women of reproductive age in Vietnam. Eur J Clin Nutr. 2015;69(1):107-13. doi: <https://doi.org/10.1038/ejcn.2014.181>.

21. National Institute of Nutrition. A review of the nutrition situation in Vietnam 2009-2010. Hanoi: National Institute of Nutrition - United Nations Children's Fund, 2011.

22. Munisi DZ, Buza J, Mpolya EA, Kinung’hi SM. Schistosoma mansoni Infections, Undernutrition and Anaemia among Primary Schoolchildren in Two Onshore Villages in Rorya District, North-Western Tanzania. PLoS One. 2016;11(12):e0167122. doi: <https://doi.org/10.1371/journal.pone.0167122>.

23. Salim N, Schindler T, Abdul U, Rothen J, Genton B, Lweno O, et al. Enterobiasis and strongyloidiasis and associated co-infections and morbidity markers in infants, preschool- and school-aged children from rural coastal Tanzania: a cross-sectional study. BMC Infect Dis. 2014;14:644. doi: <https://doi.org/10.1186/s12879-014-0644-7>. PubMed PMID: PMC4271451.

24. Winkler MS, Knoblauch AM, Righetti AA, Divall MJ, Koroma MM, Fofanah I, et al. Baseline health conditions in selected communities of northern Sierra Leone as revealed by the health impact assessment of a biofuel project. Int Health. 2014;6(3):232-41. doi: <https://doi.org/10.1093/inthealth/ihu031>.

25. Bustinduy AL, Parraga IM, Thomas CL, Mungai PL, Mutuku F, Muchiri EM, et al. Impact of Polyparasitic Infections on Anemia and Undernutrition among Kenyan Children Living in a Schistosoma haematobium-Endemic Area. Am J Trop Med Hyg. 2013;88(3):433-40. doi: <https://doi.org/10.4269/ajtmh.12-0552>. PubMed PMID: PMC3592521.

26. Casapía M, Joseph SA, Núñez C, Rahme E, Gyorkos TW. Parasite and maternal risk factors for malnutrition in preschool-age children in Belen, Peru using the new WHO Child Growth Standards. Br J Nutr. 2007;98(6):1259-66. Epub 12/01. doi: <https://doi.org/10.1017/S0007114507795272>.

27. Fernando SD, Paranavitane SR, Rajakaruna J, Weerasinghe S, De Silva D, Wickremasinghe AR. The health and nutritional status of school children in two rural communities in Sri Lanka. Trop Med Int Health. 2000;5(6):450-2. doi: <https://doi.org/10.1046/j.1365-3156.2000.00569.x>.

28. WHO. WHO methods and data sources for global burden of disease estimates 2000-2015. Geneva: World Health Organization, 2017.

29. Humphries D, Mosites E, Otchere J, Twum WA, Woo L, Jones-Sanpei H, et al. Epidemiology of Hookworm Infection in Kintampo North Municipality, Ghana: Patterns of Malaria Coinfection, Anemia, and Albendazole Treatment Failure. The American Journal of Tropical Medicine and Hygiene. 2011;84(5):792-800. doi: 10.4269/ajtmh.2011.11-0003. PubMed PMID: PMC3083749.

30. Mboera LEG, Senkoro KP, Rumisha SF, Mayala BK, Shayo EH, Mlozi MRS. Plasmodium falciparum and helminth coinfections among schoolchildren in relation to agro-ecosystems in Mvomero District, Tanzania. Acta Trop. 2011;120(1):95-102. doi: <https://doi.org/10.1016/j.actatropica.2011.06.007>.

31. Yimam Y, Degarege A, Erko B. Effect of anthelminthic treatment on helminth infection and related anaemia among school-age children in northwestern Ethiopia. BMC Infect Dis. 2016;16:613. doi: 10.1186/s12879-016-1956-6. PubMed PMID: PMC5084399.

32. Anah MU, Ikpeme OE, Etuk IS, Yong KE, Ibanga I, Asuquo BE. Worm Infestation And Anaemia Among Pre-school Children Of Peasant Farmers In Calabar, Nigeria. Niger J Clin Pract. 2008;11(3):220-4.

33. Le HT, Brouwer ID, Verhoef H, Nguyen KC, Kok FJ. Anemia and intestinal parasite infection in school children in rural Vietnam. Asia Pac J Clin Nutr. 2007;16(4):716-23.

34. Nkuo-Akenji TK, Chi PC, Cho JF, Ndamukong KKJ, Sumbele I. Malaria and helminth co-infection in children living in a malaria endemic setting of mount Cameroon and predictors of anemia. J Parasitol. 2006;92(6):1191-5. doi: 10.1645/GE-895R.1.

35. Ahmed A, Al-Mekhlafi HM, Al-Adhroey AH, Ithoi I, Abdulsalam AM, Surin J. The nutritional impacts of soil-transmitted helminths infections among Orang Asli schoolchildren in rural Malaysia. Parasites & Vectors. 2012;5:119-. doi: 10.1186/1756-3305-5-119. PubMed PMID: PMC3419660.

36. Thomas M, Woodfield G, Moses C, Amos G. Soil-transmitted helminth infection, skin infection, anaemia, and growth retardation in schoolchildren of Taveuni Island, Fiji. N Z Med J. 2005;118(1216).
